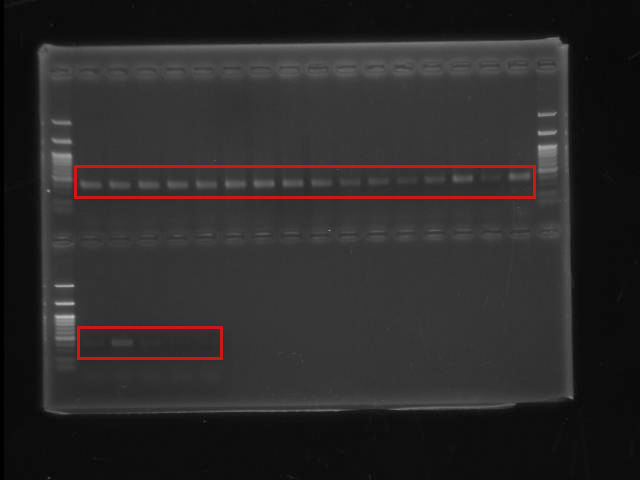# Unknown genes, *Cebelin* and *Cebelin-like*, predominantly expressed in mouse brain

**DOI:** 10.1016/j.heliyon.2018.e00773

**Published:** 2018-09-06

**Authors:** Hiroyuki Miwa, Nobuyuki Itoh

**Affiliations:** Department of Genetic Biochemistry, Kyoto University Graduate School of Pharmaceutical Sciences, Sakyo, Kyoto 606-8501, Japan

**Keywords:** Developmental biology, Biochemistry, Molecular biology, Neuroscience

## Abstract

We identified two genes, *Cebelin* and *Cebelin-like*, encoding unknown proteins in mice. Cebelin and Cebelin-like consist of 168 and 167 amino acids with putative secreted signal sequences. However, Cebelin and Cebelin-like are cellular proteins not secreted proteins. *Cebelin* and *Cebelin-like* were predominantly expressed in the brain among major tissues examined. The expression of *Cebelin* in the brain was predominantly detected in the internal granule layer of the cerebellum.

## 1 Introduction

Proteins with putative secreted signal sequences are mostly secreted or membrane proteins. Secreted proteins potentially play crucial roles as extracellular signaling molecules in cell proliferation, differentiation, and function. The identification and characterization of unknown genes encoding secreted proteins potentially provide new insights into morphogenesis, metabolism, and disease (Klee et al., 2004; Kassai et al., 2005; Wakahara et al., 2007; Koike et al., 2007; Miwa et al., 2009; Miyake et al., 2009; Ohta et al., 2015). Additionally, genes expressed by specific cells could become useful markers in developmental biology (Miwa and Era, 2015, 2016, 2018). We identified mouse cDNAs encoding unknown proteins with putative secreted signal sequences but not putative transmembrane domains from GenBank. We termed one of them *Cebelin*, which is also referred to as *Fam163a*, as the gene was predominantly expressed in the cerebellum.

## 2 Results and discussion

**Fig. 1:**
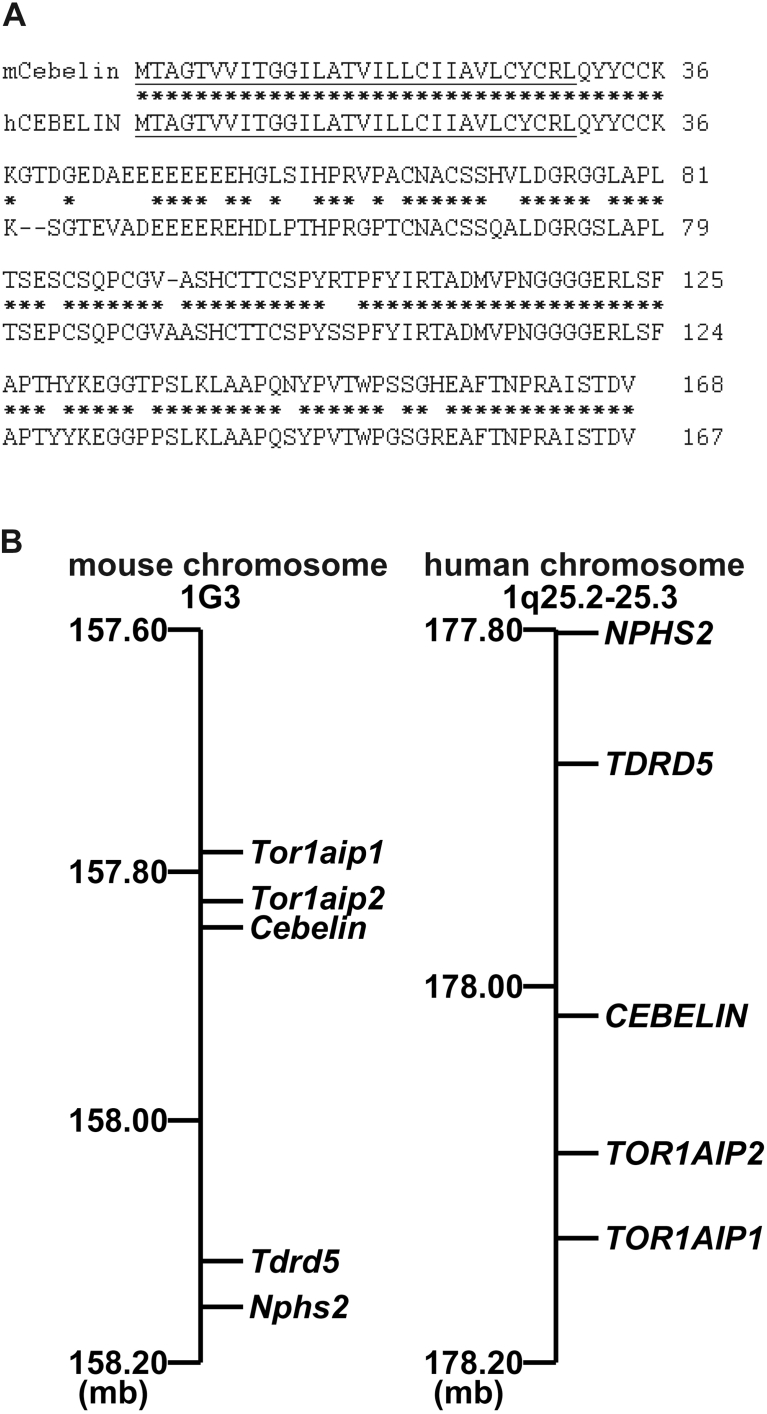
Molecular analysis of *Cebelin*. A: Comparison of AA sequences of mouse Cebelin and human CEBELIN. The numbers refer to AA positions of mouse Cebelin and human CEBELIN. Asterisks represent identical residues of the sequences. Underlines represent putative secreted signal sequences. Dashes represent introduced gaps to align the sequences. B: Syntenic relationship between mouse chromosome 1G3 and human chromosome 1q25.2-25.3. The mouse *Cebelin* and human *CEBELIN* genes are closely linked to the mouse *Tor1aip1*, *Tor1aip2*, *Tdrd5*, or *Nphs2* genes and human *TORLAIP1*, *TORLAIP2*, *TDRD5*, or *NPHS2* genes, respectively. mb, megabase.

The full-length cDNA was cloned by polymerase chain reaction (PCR) with mouse brain cDNA as a template. Cebelin protein consists of 168 amino acids (AAs) with a putative secreted signal sequence (30 AAs) at its amino terminus but not putative transmembrane domains (GenBank accession code NM_177838) (Fig. 1A). Cebelin is a unique protein with no known functional motifs and no primary structure similarity to known functional proteins. Human *CEBELIN* cDNA was also identified by a homology-based search from GenBank. The AA sequence of human CEBELIN (167 AAs) with a putative secreted signal sequence (30 AAs) was highly similar (∼85% AA identity) to that of mouse Cebelin (Fig. 1A). The coding region of *Cebelin* is divided with a single intron (data not shown). Mouse *Cebelin* is closely linked to *Tor1aip1*, *Toriaip2*, *Tdrd5* and *Nphs2* on chromosome 1 at G3. Human *CEBELIN* is also closely linked to these genes on chromosome 1 at q25.2-25.3, supporting that human *CEBELIN* is a human ortholog of mouse *Cebelin* (Fig. 1B).

**Fig. 2:**
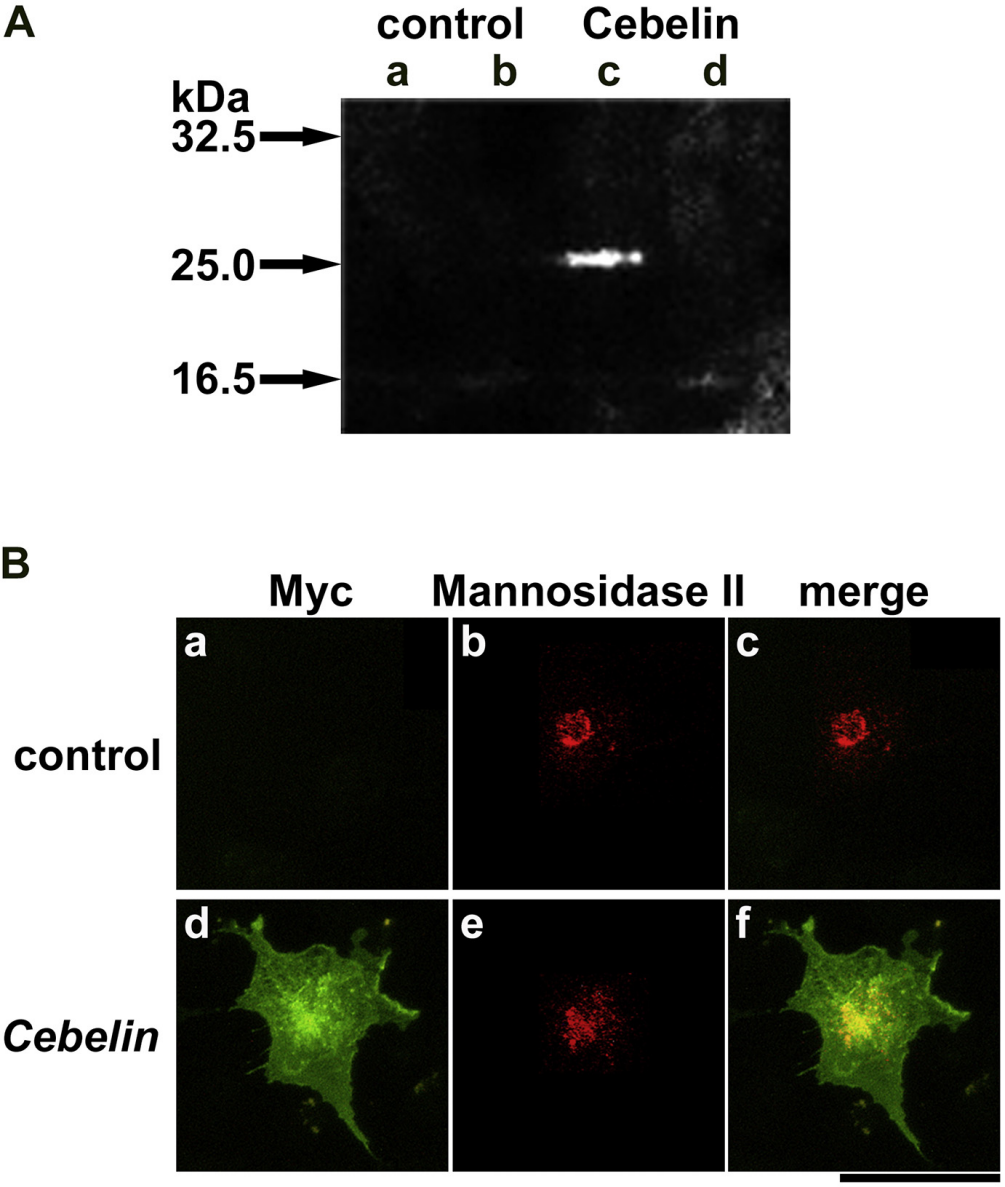
Detection of recombinant Cebelin. A: COS-7 cells were transfected with the empty vector (control) (a, b) or the recombinant Cebelin-expression vector (Cebelin) (c, d). The lysate (a, c) and medium (b, d) of the transfected COS-7 cells were examined by Western blotting using anti-Myc tag antibody. Fig. S1 is a full image of the blot. B: COS-7 cells transfected with the empty vector (control) (a–c) or the recombinant Cebelin-expression vector (*Cebelin*) (d–f) were examined by immunocytochemical using anti-Myc tag antibody for the recombinant Cebelin protein and anti-Mannosidase II antibody for the Golgi apparatus. The signals obtained by immunocytochemical using anti-Myc tag antibody (a, d) and anti-Mannosidase II antibody (b, e) were merged (c, f). Scale bar = 50 μm.

To examine whether Cebelin is a secreted protein, Myc and His_6_ tags-fused Cebelin was overexpressed in mammalian cells, COS-7 cells. Both the medium and lysate of the cultured cells were examined by Western blotting using anti-Myc tag antibody. We could detect no bands in the medium or lysate of the control. A band was detected in the lysate but not the medium of the *Cebelin*-overexpressed cells, indicating that Cebelin is a cellular protein but not a secreted protein (Fig. 2A). This result was discrepant from the previous study (Vasudevan et al., 2009). The observed molecular mass (∼25 kDa) was larger than the calculated molecular mass of the recombinant Cebelin protein (∼20.5 kDa), indicating that Cebelin protein might be subjected to post-translational modification.

We also examined the cellular localization of Cebelin in the cells by immunocytochemical analysis using anti-Myc tag antibody. No signals were detected in the control. In contrast, Cebelin was widely detected in the *Cebelin*-overexpressed cells. Cebelin was most intensely co-localized with Mannosidase II, a marker protein for the Golgi apparatus (Moremen and Touster, 1986), indicating that Cebelin was most intensely detected in the Golgi apparatus (Fig. 2B). Cebelin is a cellular protein with a putative secreted signal sequence. As hydrophobic segments at the amino termini were reported to potentially function as type II membrane protein signal anchors (Yokoyama-Kobayashi et al., 1999), the putative secreted signal sequence in Cebelin might function as the type II signal anchor.

**Fig. 3:**
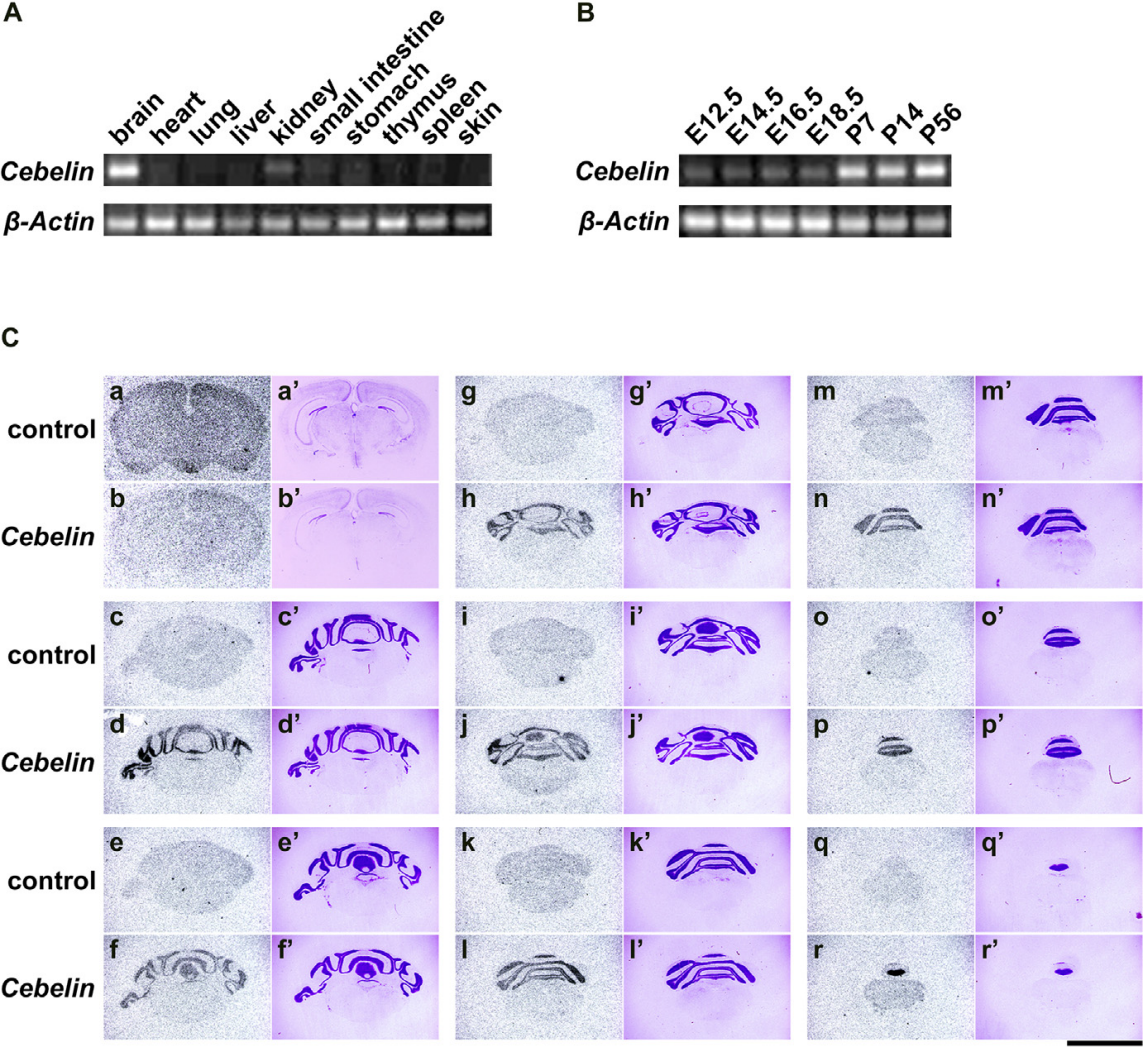
Expression of *Cebelin* in adult mouse tissues and brain at respective developmental stages, and localization of *Cebelin* in adult mouse brain. A: The expression of *Cebelin* was examined in adult mouse tissues (P56) by RT-PCR. *β-Actin* was a control. The expected sizes of *Cebelin* and *β-Actin* cDNA are 574 and 408 base pairs, respectively. Fig. S2A and B are full images of the gels. B: The expression of *Cebelin* was examined in mouse brain at respective developmental stages (E12.5-P56) by RT-PCR. C: The localization of *Cebelin* was examined in adult mouse brain (P56) by in situ hybridization using the sense (a, c, e, g, i, k, m, o, q) or antisense (b, d, f, h, j, l, n, p, r) *Cebelin* RNA probe. Black grains show the location of *Cebelin*. The sections of the brain were counterstained with cresyl-violet (a′–r′). Scale bars = 5 mm.

The expression of *Cebelin* was examined in adult mouse tissues (postnatal day 56, P56) by reverse transcription (RT)-PCR using the specific primers for *Cebelin*. Although all the tissues examined expressed *β-Actin* (Tokunaga et al., 1986), the expression of *Cebelin* was predominantly detected in the brain (Fig. 3A). We also examined the expression of *Cebelin* in the brain at respective developmental stages (embryonic day 12.5, E12.5-P56). The expression of *Cebelin* was more abundantly detected in the postnatal brain than the embryonic brain (Fig. 3B).

The expression of *Cebelin* was also examined in the adult brain by in situ hybridization using the antisense *Cebelin* RNA probe. Essentially we could detect no grains on any sections with the sense probe as a control. In contrast, the expression of *Cebelin* shown by black grains was predominantly detected in the internal granule layer of the cerebellum with the antisense probe (Fig. 3C). However, the expression of *Cebelin* was not significantly detected in any other region of the brain.

**Fig. 4:**
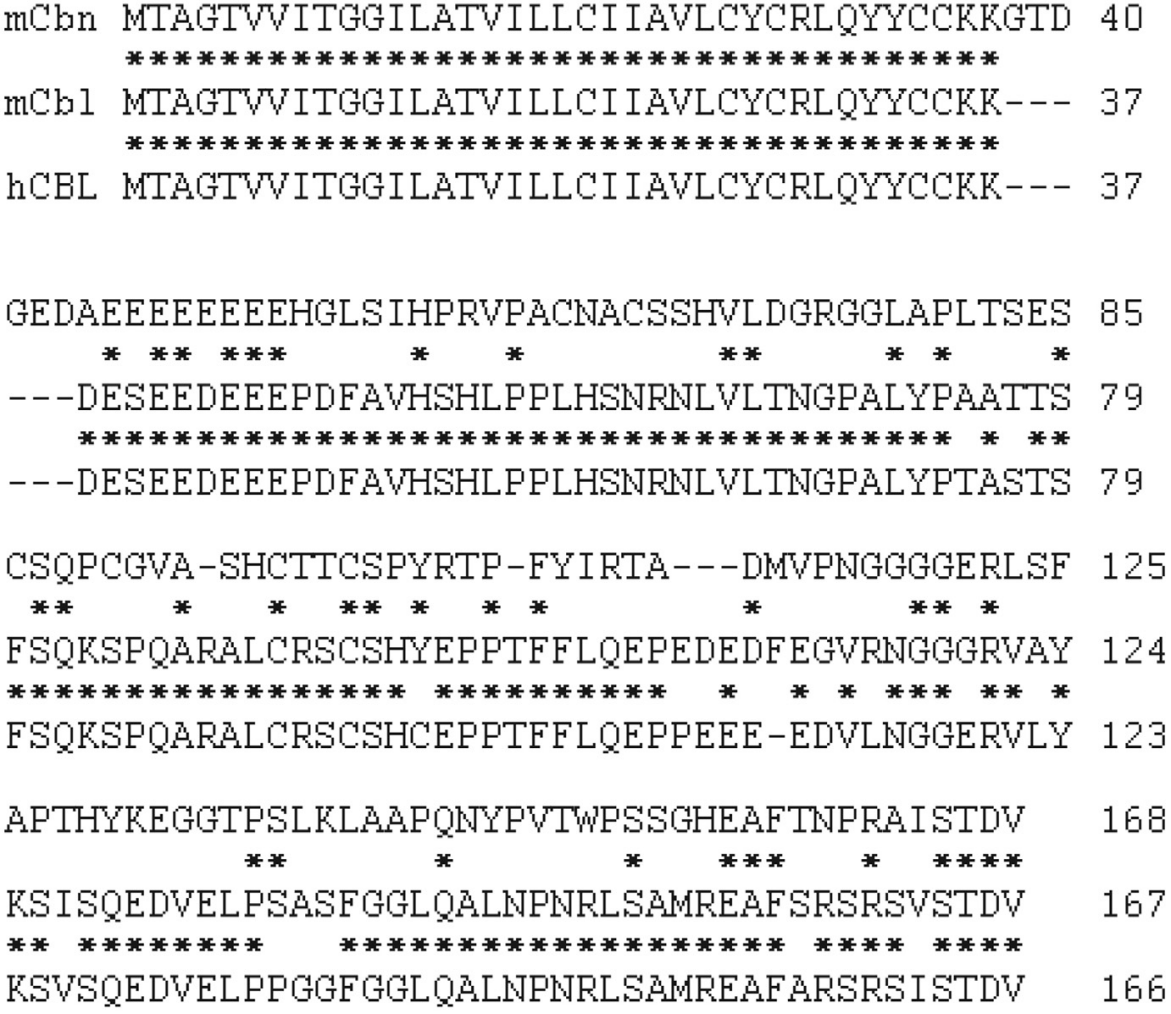
Molecular analysis of *Cebelin-like*. Comparison of AA sequences of mouse Cebelin-like (mCbl), human CEBELIN-LIKE (hCBL), and mouse Cebelin (mCbn). The numbers refer to AA positions of mouse Cebelin-like, human CEBELIN-LIKE, and mouse Cebelin. Asterisks represent identical residues of the sequences. Dashes represent introduced gaps to align the sequences.

Furthermore, we identified mouse cDNA encoding another unknown protein of 167 AAs (GenBank accession code NM_175427) (Fig. 4). As the protein is significantly similar (∼43% AA identity) to Cebelin, we named it Cebelin-like, which is also referred to as *Fam163b*. Human *CEBELIN-LIKE* cDNA was also identified. The AA sequence of human CEBELIN-LIKE (166 AAs) was highly similar (∼90% identity) to that of mouse Cebelin-like (Fig. 4).

**Fig. 5:**
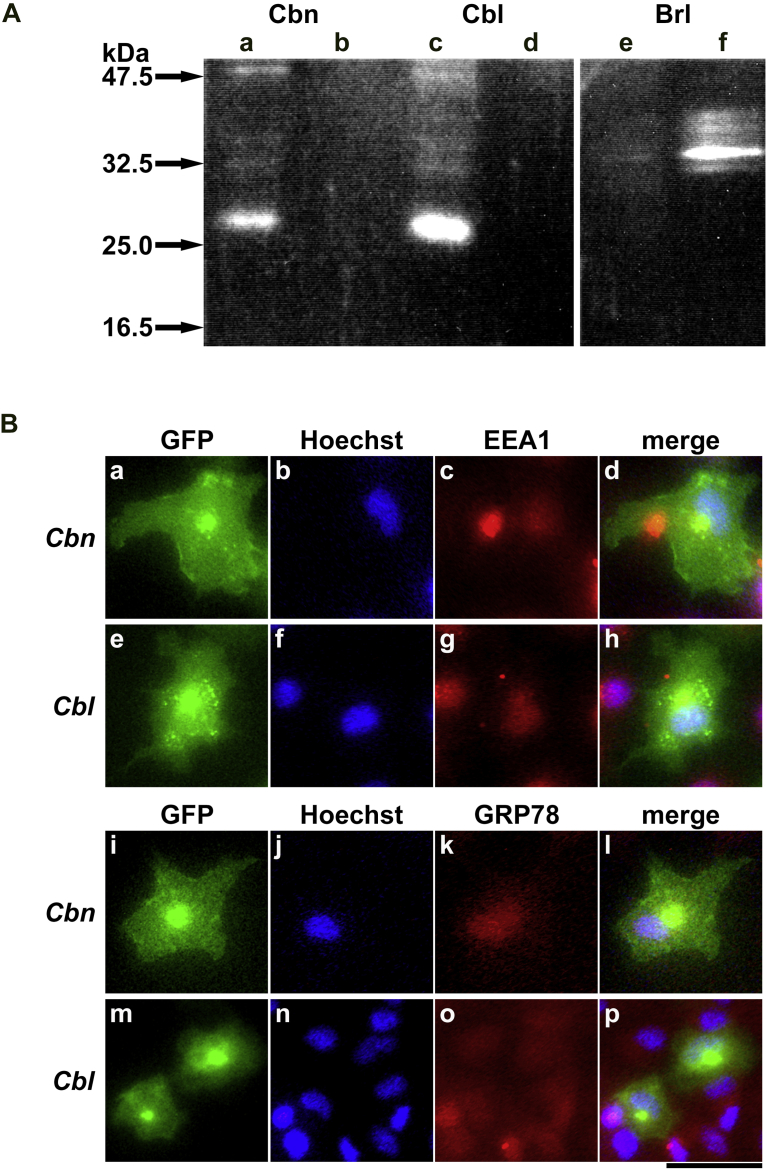
Detection of recombinant Cebelin-like. A: CHO-S cells were transfected with the recombinant Cebelin-expression vector (Cbn) (a, b), the recombinant Cebelin-like-expression vector (Cbl) (c, d) or the recombinant Brorin-like-expression vector (Brl) (e, f), which was a control. The lysate (a, c, e) and medium (b, d, f) of the transfected CHO-S cells were examined by Western blotting using anti-Myc tag antibody. Fig. S3A and B are full images of the blots. B: COS-7 cells transfected with the GFP-fused Cebelin-expression vector (*Cbn*) (a–d, i–l) or the GFP-fused Cebelin-like-expression vector (*Cbl*) (e–h, m–p) were examined by immunocytochemical using anti-EEA1 antibody for the endosome or anti-GRP78 antibody for the endoplasmic reticulum. The signals obtained by GFP (a, e, i, m), Hoechst (b, f, j, n), and immunocytochemical using anti-EEA1 antibody (c, g) or anti-GRP78 antibody (k, o) were merged (d, h, l, p). Scale bar = 50 μm.

Cebelin-like was overexpressed in CHO-S cells in the same way as Cebelin was. Both the medium and lysate of the cultured cells were examined by Western blotting. The result indicates that Cebelin-like is also a cellular protein, whereas Brorin-like is a secreted protein as described previously (Miwa et al., 2009) (Fig. 5A).

To examine the cellular localization of Cebelin-like in the cells, a green fluorescent protein (GFP)-fused Cebelin-like was overexpressed in COS-7 cells. In the result, the localization of Cebelin-like was similar to that of Cebelin and only partly overlapped EEA1, a marker protein for the endosome (Mu et al., 1995), or GRP78, the endoplasmic reticulum (Kozutsumi et al., 1988) (Fig. 5B).

**Fig. 6:**
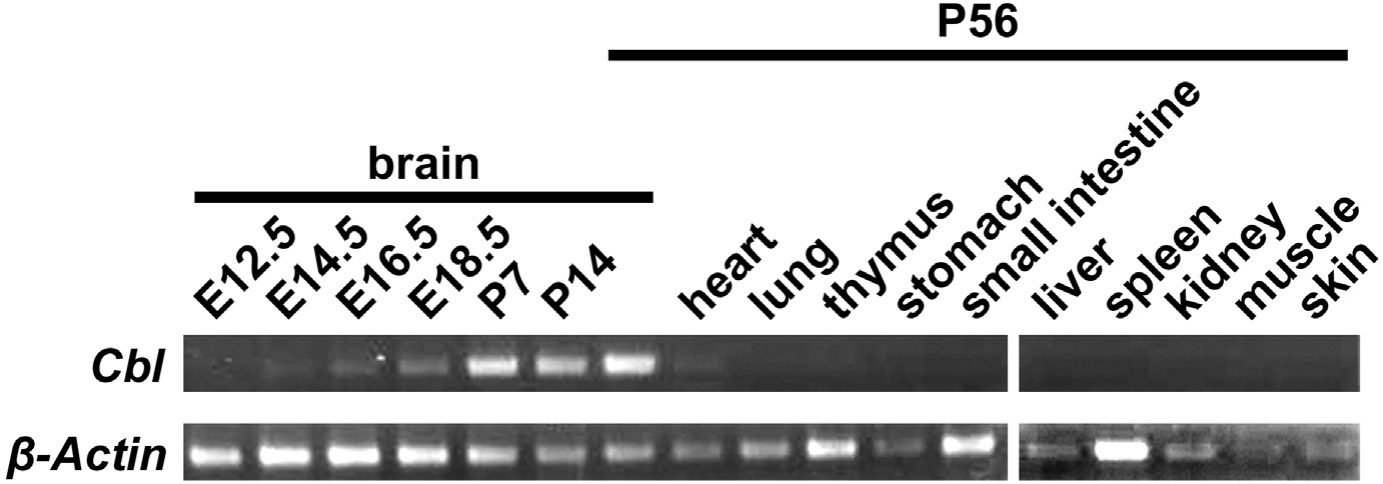
Expression of *Cebelin-like* in adult mouse tissues and brain at respective developmental stages. The expression of *Cebelin-like* was examined in adult mouse tissues (P56) and brain at respective developmental stages (E12.5-P56) by RT-PCR. *β-Actin* was a control. The expected sizes of *Cebelin-like* and *β-Actin* cDNA are 543 and 408 base pairs, respectively. Fig. S4A and B are full images of the gels.

The expression of *Cebelin-like* was examined in the embryonic brains and adult tissues by RT-PCR. The expression profiles of *Cebelin-like* are also similar to those of *Cebelin* (Fig. 6).

In conclusion, we identified two genes, *Cebelin* and *Cebelin-like*, encoding unknown proteins in mice and human. Both Cebelin and Cebelin-like are cellular proteins not secreted proteins and predominantly expressed in the brain. The present findings indicate that *Cebelin* and *Cebelin-like* are unknown genes encoding cellular proteins that potentially play roles in the cerebellum.

## 3 Experimental

### 3.1 Mice

The Animal Research Committee of Kyoto University Graduate School of Pharmaceutical Sciences approved all study protocols. All mice were purchased from Shimizu Laboratory Supplies.

### 3.2 Identification of Cebelin and Cebelin-like in mice and humans

AA sequences predicted from mouse cDNAs of unknown function in nucleotide sequence databases were randomly analyzed using PSORT. The cDNAs encoding putative secreted proteins were identified and cloned in pGEM-T Easy vector (Promega). We named two of the cDNAs mouse *Cebelin* and *Cebelin-like*. Human *CEBELIN* or *CEBELIN-LIKE* cDNA was also identified in a homology-based search of human cDNA sequences in nucleotide sequence databases with the AA sequence of mouse Cebelin or Cebelin-like.

### 3.3 Forced expression of Cebelin or Cebelin-like cDNA in COS-7 cells and CHO-S cells

The *Cebelin* or *Cebelin-like* cDNA with a DNA fragment encoding a Myc tag and a His_6_ tag or a GFP at the 3′ terminus of the coding region was constructed in pcDNA3.1(+) vector (Thermo Fisher Scientific).

COS-7 cells and CHO-S cells were transfected with the respective vectors using Lipofectamine 2000 (Thermo Fisher Scientific) and cultured at 37 °C in a humidified atmosphere of 5% CO_2_ in air.

### 3.4 Detection of recombinant Cebelin or Cebelin-like protein

For Western blotting, the samples were separated by sodium dodecyl sulfate-polyacrylamide gel electrophoresis (SDS-PAGE) under reducing conditions and transferred onto Hybond-ECL (GE Healthcare). The recombinant proteins were detected using mouse monoclonal anti-Myc tag antibody (Cell Signaling Technology) (1:500) as primary antibody and HRP-conjugated rabbit anti-mouse IgG antibody (Thermo Fisher Scientific) (1:1,000) as secondary antibody. Immunoreactive bands were visualized using an enhanced chemiluminescence detection system (PerkinElmer) as described (Yamashita et al., 2002).

To detect Cebelin by immunocytochemical analysis, mouse monoclonal anti-Myc tag antibody and FITC-conjugated goat anti-mouse IgG (Sigma-Aldrich) were used as primary and secondary antibodies, respectively. To detect Mannosidase II, EEA1, and GRP78, rabbit anti-Mannosidase II antibody, anti-EEA1 antibody, and anti-GRP78 antibody (Abcam) and TRITC-conjugated goat anti-rabbit antibody (Sigma-Aldrich) were used as primary and secondary antibodies, respectively.

### 3.5 RT-PCR

Total RNA was purified with RNeasy Mini kit (Qiagen) and transcribed to DNA using M-MLV Reverse Transcriptase (Thermo Fisher Scientific). The cDNAs were amplified with Gene *Taq* NT (Nippon Gene) and the specific primers, which were listed in Table 1. DNA fragments were detected by agarose gel electrophoresis.

**Table 1:** Primers for RT-PCR

Gene	Sequence (forward)	Sequence (reverse)
*Cebelin*	5′-ATACATCTTTGCAGAGTTTGATGG-3′	5′-TGTTGGGTGTGTGCAGATTGG-3′
*Cebelin-like*	5′-AGGCTGTTGATGGAGAAGTGG-3′	5′-AGGATAGAGGCCTGTCACACG-3′
*β-Actin*	5′-CAGAGCAAGAGAGGTATCCT-3′	5′-CGGTCAGGATCTTCATGAGG-3′

### 3.6 In situ hybridization

Mouse brain at P56 was frozen in O.C.T. compound (Sakura Finetek), and sections were cut at 10 μm. A ^35^S-labeled sense or antisense RNA probe was transcribed from *Cebelin* cDNA clone. The signals were visualized by autoradiography using BioMax MR (Carestream) as described (Yazaki et al., 1994). The sections of mouse brain were counterstained with cresyl-violet (Nissl staining).

## Declarations

### Author Contribution statement

Hiroyuki Miwa: Conceived and designed the experiments; Performed the experiments; Analyzed and interpreted the data; Contributed reagents, materials, analysis tools or data; Wrote the paper.

Nobuyuki Itoh: Conceived and designed the experiments; Analyzed and interpreted the data; Contributed reagents, materials, analysis tools or data; Wrote the paper.

### Funding statement

This research did not receive any specific grant from funding agencies in the public, commercial, or not-for-profit sectors.

### Competing interest statement

The authors declare no conflict of interest.

### Additional information

No additional information is available for this paper.

## Figures and Tables

**Fig.S1:**
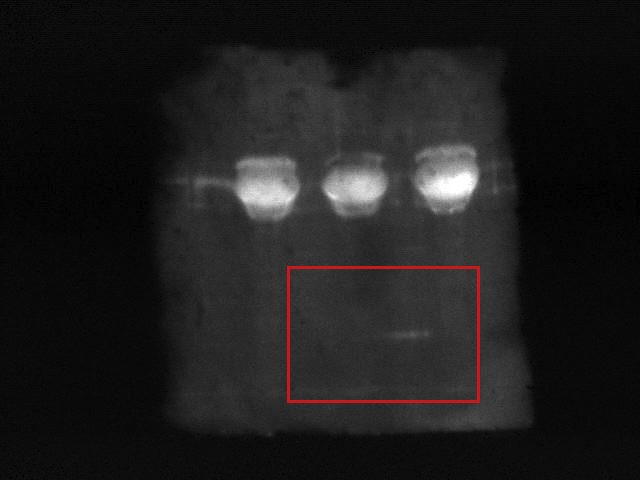


**Fig.S2A:**
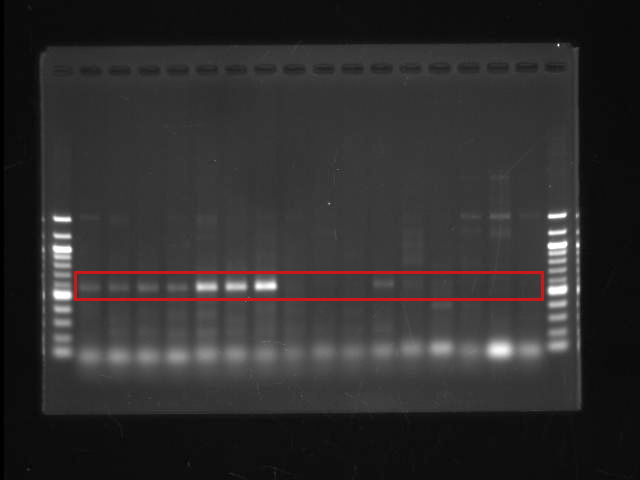


**Fig.S2B:**
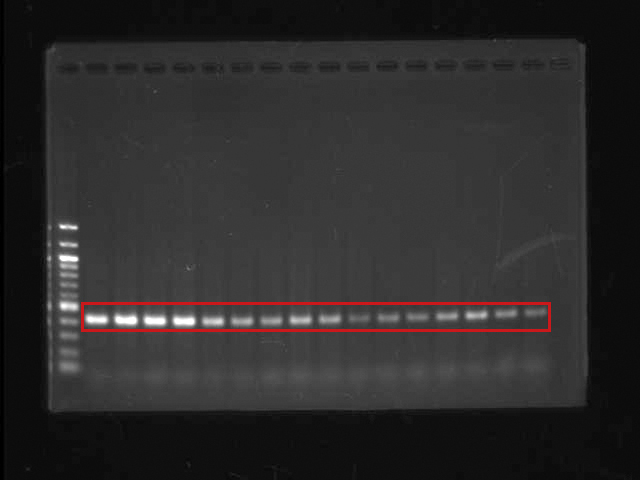


**Fig.S3A:**
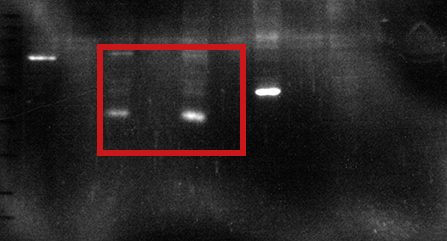


**Fig.S3B:**
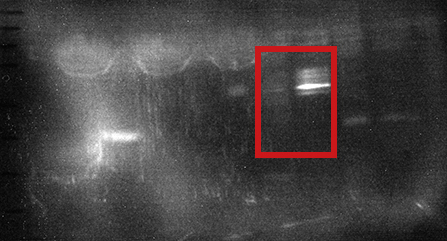


**Fig.S4A:**
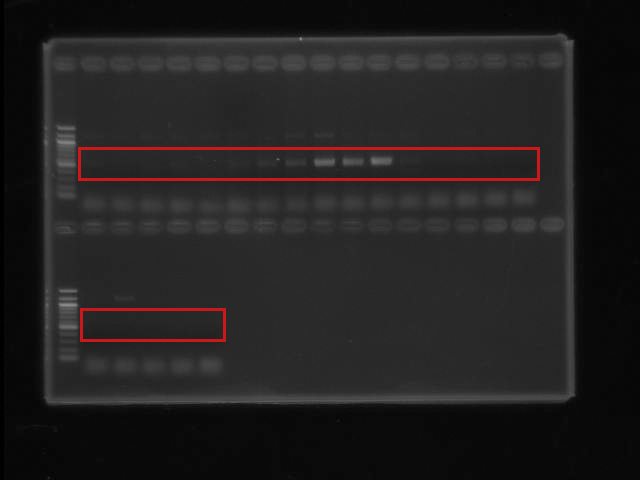


**Fig.S4B:**